# Interventional Radiology Image-Guided Locoregional Therapies (LRTs) and Immunotherapy for the Treatment of HCC

**DOI:** 10.3390/cancers13225797

**Published:** 2021-11-18

**Authors:** Pierpaolo Biondetti, Lorenzo Saggiante, Anna Maria Ierardi, Massimo Iavarone, Angelo Sangiovanni, Filippo Pesapane, Enrico Maria Fumarola, Pietro Lampertico, Gianpaolo Carrafiello

**Affiliations:** 1 Diagnostic and Interventional Radiology Department, IRCCS Cà Granda Fondazione Ospedale Maggiore Policlinico, Università degli Studi di Milano, 20122 Milan, Italy; annamaria.ierardi@policlinico.mi.it (A.M.I.); gianpaolo.carrafiello@unimi.it (G.C.); 2 Postgraduate School in Radiodiagnostics, Università degli Studi di Milano, 20122 Milan, Italy; lorenzo.saggiante@unimi.it; 3 Gastroenterology Department, IRCCS Cà Granda Fondazione Ospedale Maggiore Policlinico, Università degli Studi di Milano, 20122 Milan, Italy; massimo.iavarone@policlinico.mi.it (M.I.); angelo.sangiovanni@policlinico.mi.it (A.S.); pietro.lampertico@unimi.it (P.L.); 4 Radiology Department, IEO European Institute of Oncology IRCCS, 20122 Milan, Italy; filippo.pesapane@ieo.it; 5 Diagnostic and Interventional Radiology Department, ASST Santi Paolo e Carlo, 20122 Milan, Italy; enrico.fumarola@asst-santipaolocarlo.it

**Keywords:** hepatocellular carcinoma, HCC, tumor immunology, locoregional therapy, ablation, radiofrequency, microwave ablation, TACE, interventional radiology, immunotherapy

## Abstract

**Simple Summary:**

Interventional radiology image-guided locoregional therapies for the treatment of HCC have demonstrated to be characterized by immunomodulatory effects on the tumoral microenvironment, and, possibly, systemic. Immunotherapy has gained an important role in the treatment of HCC over the last several years. Currently, there is great interest in combining locoregional therapies with immunotherapy, as this could open a new chapter in the history of HCC treatment. In this review, after describing the immune system changes caused by the tumor, we describe, for each locoregional therapy, technique and immunomodulatory effects. Then, we describe the current status of immunotherapy in HCC and report the ongoing clinical studies testing the combination treatment.

**Abstract:**

Image-guided locoregional therapies (LRTs) are a crucial asset in the treatment of hepatocellular carcinoma (HCC), which has proven to be characterized by an impaired antitumor immune status. LRTs not only directly destroy tumor cells but also have an immunomodulating role, altering the tumor microenvironment with potential systemic effects. Nevertheless, the immune activation against HCC induced by LRTs is not strong enough on its own to generate a systemic significant antitumor response, and it is incapable of preventing tumor recurrence. Currently, there is great interest in the possibility of combining LRTs with immunotherapy for HCC, as this combination may result in a mutually beneficial and synergistic relationship. On the one hand, immunotherapy could amplify and prolong the antitumoral immune response of LRTs, reducing recurrence cases and improving outcome. On the other hand, LTRs counteract the typical immunosuppressive HCC microenvironment and status and could therefore enhance the efficacy of immunotherapy. Here, after reviewing the current therapeutic options for HCC, we focus on LRTs, describing for each of them the technique and data on its effect on the immune system. Then, we describe the current status of immunotherapy and finally report the recently published and ongoing clinical studies testing this combination.

## 1. Hepatocellular Carcinoma and Its Current Therapeutic Options 

Liver cancer is the sixth most commonly diagnosed cancer and the third leading cause of cancer death worldwide, after lung and colorectal cancer. In 2020, an estimated age-standardized incidence rate of 905,677 new liver cancers were reported, accounting for 830,180 deaths per year [1]. Hepatocellular carcinoma (HCC) represents approximately 75–85% of primary liver cancers [2]. The worldwide incidence of HCC is heterogeneous, depending on the distribution of risk factors, above all hepatitis B, hepatitis C, alcohol and metabolic syndrome [3]. Whatever the etiology, cirrhosis is the main risk factor for liver cancer development; HCC is associated with cirrhosis in more than 80% of cases, and it has been estimated that one-third of cirrhotic patients will develop liver cancer during their lifetime [4,5] with a 1–8% annual incidence reported in long-term follow-up studies. 

The choice of treatment modality for HCC depends on several variables such as the extent of liver cancer, severity of the underlying chronic liver disease, presence of comorbidities, and performance status. Therapy must be tailored after a careful evaluation of patients, which can only be achieved by a multidisciplinary team, including a hepatologist, an oncologist, a transplant-hepatobiliary surgeon, a radiologist, an interventional radiologist, and a pathologist, following recommendations provided by the International Scientific Societies. Although there are discrepancies among the International Scientific Societies’ recommendations, there is a consensus that radical treatments, namely, liver transplantation, surgical resection, and locoregional treatments (LRTs) (i.e., radiofrequency ablation (RFA) and microwave ablation (MWA)) are indicated for early-stage HCC, which includes monofocal HCC and multifocal HCC with no more than three nodules, no larger than 3 cm each; trans-arterial chemoembolization (TACE) for intermediate-stage HCC; systemic therapy for advanced HCC. In detail, according to EASL-EORTC guidelines [6], the recommended first-line treatments are RFA or MWA for early monofocal HCC up to 2 cm in size and for multifocal HCC with up to three nodules, no larger than 3 cm, while surgery is recommended for monofocal tumors exceeding 2 cm. Liver transplantation is recommended for de novo multifocal tumor or recurrent HCC within “Milan criteria” (i.e., single nodules less than 5 cm in diameter or up to 3 nodules less than 3 cm each). For larger HCCs, classified in the intermediate stage, TACE is the treatment of choice, while systemic therapy is dispensed to patients with advanced stage, which includes patients with macrovascular invasion or metastatic HCC. Additional treatment options are under investigation such as cryoablation for early HCC and trans-arterial radioembolization (TARE) for advanced HCC with neoplastic portal invasion and no extrahepatic metastases.

Much more complex and less defined are further lines of treatment for patients showing no complete response to first-line therapies. Many studies have tested the ability to improve survival combining the abovementioned treatments as well as neoadjuvant or adjuvant therapies. Unfortunately, all these approaches have failed to improve overall survival (OS), so that none of them are recommended by the Scientific Societies.

In recent years, a significant improvement in OS has been obtained with systemic treatments. Many targeted therapies tested in randomized controlled trials obtained a survival improvement and have received FDA and EMA approval including tyrosine kinase inhibitors (TKIs) and anti-vascular endothelial growth factor (anti-VEGFR), such as sorafenib and lenvatinib as first-line treatments and regorafenib, ramucirumab, and cabozantinib as second-line treatments. Sorafenib is approved for patients with Child–Pugh (CP) A, while few prospective data are available for CP B patients, with the use of sorafenib being reserved in practice for patients with advanced HCC with compensated CP B [7]. As for lenvatinib, also approved for HCC patients with CP A liver function, it has been suggested that it may be continued in patients whose liver function deteriorates to CP B [8]. Few data are available on regorafenib in CP B patients, although a recent study advises against its use in this population, given the high frequency of adverse events and the poor clinical outcomes [9]; similar results were found regarding ramucirumab [10]. In the phase III CELESTIAL study (NCT01908426), cabozantinib, compared to the placebo, showed a manageable safety profile and clinical benefit.

More recently, immune checkpoint inhibitor (ICI) molecules, such as nivolumab and pembrolizumab, emerged as potential treatment options for HCC. Finally, the combination of two different targeted therapies emerged as a successful strategy, as the combination of the programmed cell-death 1 ligand 1 (PD-L1) inhibitor atezolizumab together with the anti-VEGF bevacizumab turned out to have significantly better outcomes compared to sorafenib as first-line treatments [11]. Thus, the combination of atezolizumab with bevacizumab is now the standard of care for the first-line treatment of advanced HCC.

The impressive benefit provided by immunotherapy leads to the question if there is a rationale to support the combination of surgery or LRTs for early or intermediate HCC with immunotherapy, given the established effect of this therapy and its modest side effects, with the possibility of shifting immunotherapy from an advanced treatment setting to an adjuvant setting, considering that it does not require hepatic metabolism and that HCC is regarded as an immunogenic cancer [12]. Several aspects of the mechanism of action of ICIs together with the tumoral cell death induced by LRTs (such as ablation or endovascular procedures) make the association of these different treatments intriguing as potentially being able to increase their mutual effectiveness on HCC.

Here, we review the rationale supporting the combination of immunotherapy with LRTs and summarize the recently published and ongoing clinical studies testing this combination.

## 2. Immune System Changes in HCC Patients

HCC develops in an altered and complex microenvironment, at the same time immune activating and immune suppressive, which can lead either to tumor eradication or to tumor progression [13]. On the one hand, HCC has been reported to be frequently recognized by the immune system [14] with various spontaneous immune responses, both humoral and T-cell mediated, to tumor-associated antigens (TAAs) [15,16]. On the other hand, there is a strong evidence that HCC is associated with an impaired antitumor immune status, both in tumor local tissue and in peripheral blood, leading to a high immune evasion [17]. Tumor regions were found to have an increased infiltration of regulatory T cells (Tregs) by 87% [18] and a reduced infiltration of CD8+ T cells, which also showed a lower expression of granzyme A (GrA), granzyme B (GrB), and perforin [19]. High Treg counts and low CD8+ T-cell counts in HCC tissues were correlated with an increase in tumor size [20,21] and with a poor prognosis [22,23,24]. Circulating Tregs were also found to increase by 66% in HCC patients [25], with an existing correlation between Treg percentage and HCC stage [26,27,28]. Furthermore, HCC patients showed a decrease in proinflammatory cytokines, such as tumor necrosis factor-α (TNF-α), interferon-γ (IFN-γ), interleukin (IL)-1, and an increase in immunosuppressive cytokines (IL-4, IL-5, IL-8, and IL-10) in the tumor microenvironment, resulting in a poor prognosis [29,30]. In addition, a high neutrophil/lymphocyte ratio was validated as a poor prognostic indicator after treatment of HCC, suggesting how HCC recurrence after liver transplant may be influenced by the inflammatory microenvironment [31].

The promising role of immunotherapy derives precisely from its potential of tipping this precarious balance between immune-stimulating and immune-suppressive status towards the former, eliciting an antitumor response. At the same time, the immune suppressive microenvironment of HCC represents a strong obstacle to the efficacy of immunotherapy. Therefore, approaches that counteract this unfavorable tumor microenvironment are needed in combination with immunotherapy. The key element in this impaired antitumor immune microenvironment is the low expression and availability of tumor antigens on cancer cells, resulting in lower T-cell activation and tumor infiltration; LRTs may play a pivotal role in overcoming this problem.

HCC is not the only tumor in which immunotherapy has proven to be beneficial; in fact, the efficacy of immunotherapy has also been demonstrated in melanoma, renal cell carcinoma, hematological malignancies, and non-small cell lung cancer, and new evidence is emerging suggesting its potential in treating other types of malignancies [32].

## 3. LRT and Immunomodulation

LRTs have the potential of shaping tumor immunity by altering the composition of the HCC microenvironment; in particular, they lead to the release from dying tumor cells of cryptic TAAs and tumor neoantigens that become accessible to the immune system, acting as novel targets for antigen-presenting cells (APCs), mainly dendritic cells (DCs).

The innate immune system, including neutrophils, macrophages, and natural killer (NK) cells, is the first to respond, followed by the more strong and sustained acquired response [33,34], characterized by significant intratumoral immune infiltrates [35,36,37,38,39,40]. In order to stimulate the acquired immune system, cancer cells must die thorough immunogenic cell death (ICD), which happens with necrosis and is characterized by the release from dying cells, along with antigens and preserved intracellular organelles, of damage-associated molecular patterns (DAMPs), such as DNA, RNA, heat shock proteins (HSPs), adenosine triphosphate (ATP), protein high-mobility group box 1 (HMGB1), calreticulin, and uric acid, which allow recruitment and activation of DCs (through the NF-κβ pathway activation). Activated DCs, after reaching regional lymph nodes, present antigens along with costimulatory signals to T cells that are therefore stimulated [41,42,43]. On the contrary, in non-ICD, which occurs with apoptosis, the dying tumor cells do not release DAMPs; without phagocytizing DAMPs, DCs cannot be activated and therefore do not present costimulatory signals to T cells [43,44], with the subsequent T-cell anergy and deletion leading to immune tolerance [45,46,47]. The antitumor response triggered by ICD takes place both locally and distantly from the primary tumor; this phenomenon, whereby a locally applied therapy elicits a distant antitumor response, is known as the abscopal effect [48].

It is not yet clear which ablative technique has the highest potential for releasing cryptic tumor antigens and creating the best immunostimulatory microenvironment.

## 4. Pro-Oncogenic Effects of LRTs

All locoregional and surgical approaches might also have a stimulatory effect of oncogenesis, as described by several studies, by means of tumor cell spreading, microenvironmental changes, and both angiogenetic and proliferative triggers.

In fact, LRTs, both thermal and non-thermal, applied to the liver as well as to other organs, have been correlated to a more aggressive tumor subtype, tumor growth [49,50,51], and to a higher incidence of tumor progression [52,53]. This can be attributed to local and systemic inflammation (via an increase in IL-6, IL-8, and HSP) and to the upregulation of pro-oncogenic growth factors such as hypoxia-inducible factor-1α (HIF-1α), VEGF, hepatocyte growth factor (HGF), hepatocyte growth factor receptor (HGF-R), matrix metalloproteinases (MMPs), cluster of differentiation 147 (CD147), and mammalian target of rapamycin (mTOR) [49,54,55,56,57,58,59,60]. Moreover, local hepatic thermal ablation has been linked, in some cases, to a higher rate of distant intrahepatic tumor development and to overall worse outcomes [50,53,60,61]. This may be particularly true for sublethal–incomplete treatments [62,63,64].

## 5. LRTs: Classification

**Figure 1:**
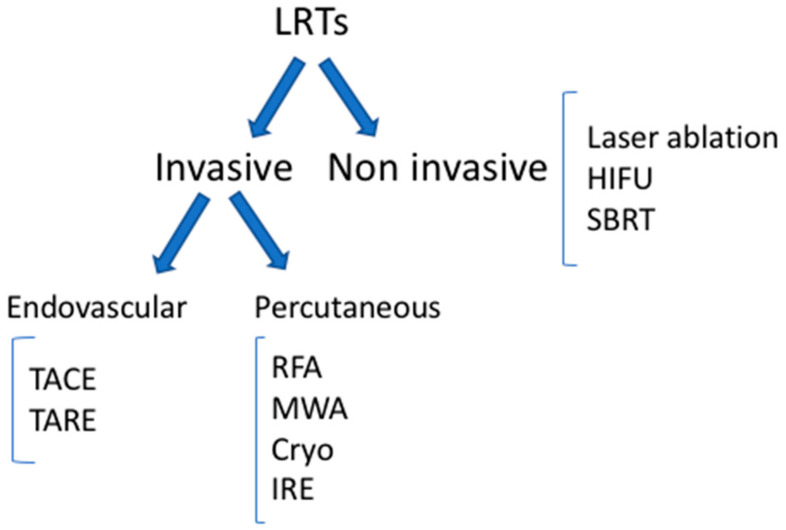
Locoregional therapies. TACE: trans-arterial chemoembolization; TARE: trans-arterial radioembolization; RFA: radiofrequency ablation; MWA: microwave ablation; IRE: irreversible electroporation; HIFU: high-intensity focal ultrasound; SBRT: stereotactic body radiation therapy.

The main types of LRTs available today are reported in Figure 1.

In the following section, we briefly describe the technical properties and methods of functioning for each LRT, together with the existing evidence regarding its effect on the immune system. In Table 1, we summarize the effect of each LRT on the immune system.

## 6. Invasive Percutaneous LRTs

### 6.1. Radiofrequency Ablation (RFA)

#### 6.1.1. Technique

RFA is the most validated and widely used technique in the early stages of HCC, and it is still considered the main ablative therapy for HCC tumors smaller than 5 cm in diameter [65,66,67]. Nevertheless, when used alone, RFA is associated with a high risk of progression and tumor recurrence, in particular for >3 cm nodules [66]. Radiofrequency waves are generated by an electrode located in a probe inserted through the skin at the tumor site under imaging guidance, typically ultrasound or CT. The electrical circuit is completed through one or more grounding pads attached to the thighs or back of the patient. A high-frequency oscillating electrical current generates heat through the frictional movement of ions, achieving temperatures between 60 and 100 °C within the central zone, which leads to coagulative necrosis of tumor cells around the electrode [45,68]. In addition, heat conduction allows sublethal temperatures to be reached in more peripheral areas, where cell death occurs by apoptosis, obtaining a larger final ablation zone. Above 100 °C, boiling and charring of tissues occur, which, by increasing impedance and decreasing electrical conduction, limit the effectiveness of RFA.

#### 6.1.2. Immunomodulation

Immune responses induced by RFA have been well documented, both local and systemic,. Following RFA of HCC, as well as of other tumors, a release of DAMPs, including RNA, DNA, HSPs, and HMGB1 [69,70], and an increase in inflammatory cytokines, such as IL-1β, IL-6, IL-8, TNF-α, and IFN-γ [34,52,71,72,73], in tumor specific antibodies, CD4+ T cells, CD8+ T cells, tumor-specific T cells [34,37,74], central memory lymphocytes (CD45RA-/CCR7+) [74,75], and in infiltrating CD45RO+ memory T cells have been demonstrated [34]. A higher count of tumor specific CD8+ T cells has been correlated with increased survival in patients with HCC [76,77] and the enhanced infiltration of CD45RO^+^ T cells is considered a marker of improved clinical outcome in all types of solid tumors [78]. Following RFA, a decrease in TGF-β, which acts as a pro-oncogenic cytokine, in IL-10, which normally stimulates tumor progression and inhibits cytotoxic T cells and NK cells [34], and in Tregs levels [34,73,79], has been reported.

The resultant increased CD8^+^ T/Treg ratio implies a shift in the immune system toward antitumor immunity. Conversely, an increased distant tumor growth following hepatic RFA has been observed, a pro-oncogenic response that may be explained by the activation of hepatocyte regeneration pathways by the injured liver parenchyma [49,50,60,80,81]. However, these data, although consistent, are based on preclinical models and do not correlate with a worse survival of RFA-treated patients compared to untreated patients [80].

A role is played by the HIF-1a/VEGF signaling pathway, which drives angiogenesis and has been shown to be upregulated in selected viable cells after exposition to heat, similar to what could happen at the margins of an incomplete ablation zone [82]. A study investigating the prognostic value of VEGF levels after RFA found that patients with higher levels of serum VEGF had a worse prognosis than patients with lower levels of serum VEGF [83].

### 6.2. Cryoablation

#### 6.2.1. Technique

Imaging-guided ablation with cryotherapy, which consists of cellular destruction by freezing, causes cellular damages through direct and vascular-mediated pathways [84]. Percutaneous cryoablation of hepatic tumor uses liquefied gases (e.g. nitrogen or argon) under high pressure, which cool down when they rapidly expand via the Joule–Thomson effect, achieving temperatures from −80 °C to just under −150 °C [85], causing mechanical stress on the cellular membranes from intracellular ice crystal formation and consequent hypotonic cell disruption [86]. Cytotoxic cell destruction is achieved at temperatures below −40 °C. Tissue ischemia also occurs due to the fact of microvascular thrombosis, which can lead to a reduction in bleeding despite the lack of cauterization that is present with RFA or MWA [87]. Depending on the tumor size, cryoablation involves the positioning of one to several probes under imaging guidance. To ensure complete ablation of the tumor, a circumferential margin of 1 cm is needed [85].

During cryoablation, the target area is imaged at 2–5 min intervals in order to monitor the area of thermal injury, given that the ice-ball is detectable with both CT and MRI, and this helps avoid damage to adjacent organs [86]. Cryoablation causes less periprocedural pain than RFA, requiring a lower dose of sedatives during the procedure [88]. A limitation of cryoablation is the increased risk of hemorrhage caused by the inability to coagulate tissue during probe withdrawal, which instead can be done with RFA or MWA [77].

#### 6.2.2. Immunomodulation

Cryoablation, leaving the ablated cancer tissue in situ, makes tumor antigens available to the host’s immune system, triggering the activation of innate and adaptive immunity against tumor antigens [89]. The immunostimulatory response following cryoablation has been described as the most potent among ablative therapies as evidenced by significantly higher post-ablative levels of serum IL-1, IL-6, NF-κβ, and TNF-α [90]. Peripheral to the site of cryoablation, sublethal temperatures induce apoptotic cell death [90]. As explained before, apoptosis may lead to T-cell anergy or clonal deletion with a consequent immunosuppressive effect [46]. Accordingly, cryoablation can induce both an immunostimulatory and an immunosuppressive effect, depending on whether there is more necrosis or apoptosis, and the proportion of these may vary over time [91].

In a murine model, TNF-α, IFN-γ, IL-1, and IL-10 were demonstrated to increase significantly after a cycle of cryoablation of tumor tissue in an experimental setting [92].

Cryoablation caused regression of untreated tumors and of lung metastases in a mouse model of prostate cancer and was associated with a systemic increase in CD4+ T cells, CD8+ T cells, and NK cells. On the contrary, cytotoxic T cells did not increase after cryoablation as opposed to heat-based ablative therapies [93,94]. Moreover, in HBV-positive HCC patients, the presence of elevated programmed cell death protein 1 (PD-1) on T cells and its relative ligand (PDL-1) on tumor cells was correlated to a poor overall survival post cryoablation [95]. However, the cryoablation-induced immune resistance with upregulation of PD-L1 on tumor cells could be overcome by combining cryoablation with a PD-1 inhibitor, such as nivolumab or pembrolizumab, which result in an effective antitumor T-cell response and in more effective disease control [96]. Finally, two clinical studies reported an increased OS when cryoablation was combined with immunotherapy, which consisted of infusion of allogenic NK cells and DC cytokine-induced killer (DC-CIK) cells [97,98]. These preliminary data show an excellent synergy between cryoablation and immunotherapies [89].

In a mice model of lung adenocarcinoma, tumor neoangiogenesis significantly increased in residual tumors with an overexpression of VEGF [99].

### 6.3. Microwave Ablation (MWA)

#### 6.3.1. Technique

**Figure 2:**
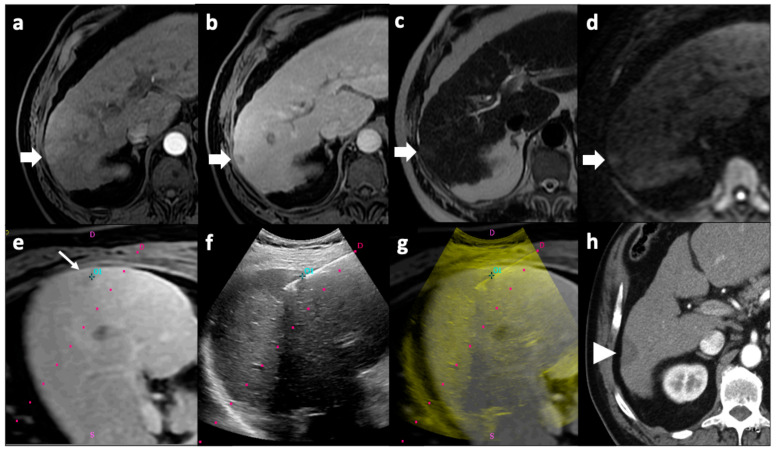
Microwave ablation of an HCC nodule. Pre-procedural MRI axial images demonstrated a focal right liver lesion with arterial wash-in (arrow, **a**), venous wash-out (arrow, **b**), hyperintensity on T2-weighted images (arrow, **c**), and diffusion restriction (arrow, **d**). The tumor was not clearly visible on US examination; thus, pre-procedural MR images in which the tumor was clearly detectable (thin arrow, **e**) were fused with real-time US images in which the microwave antenna could be monitored (**f**) during procedure; 01 corresponds to the point where the target lesion was selected on pre-procedural images. A fused MR–US image was produced showing the antenna correctly positioned inside the nodule (**g**). The red dot line in images (**e**–**g**) indicate the predicted path of the antenna when a needle guidance system is used, but in this case guidance was used only to enter liver parenchyma, not for precisely inserting the antenna inside the nodule. Follow-up axial CT imaging performed 1 month after the procedure showed a hypodense image consisting with the ablation zone (arrowhead, **h**) with no enhancement in the arterial phase, consistent with the absence of residual disease. CT: computed tomography; HCC: hepatocellular carcinoma; MRI: magnetic resonance; US: ultrasound.

MWA uses an oscillating electromagnetic energy in the microwave range (with frequencies of at least 900 MHz) to cause agitation of water molecules in the targeted tissue, resulting in frictional heat that damages cells via hyperthermic injury [100,101], leading to cell death by coagulative necrosis [102]. Microwaves are generated by an antenna which is placed directly into the target zone under imaging guidance [103]. The advantages of MWA, compared to RFA, include higher intra-tumoral temperatures, shorter application times, larger ablation areas, and possibly decreased periprocedural pain [102,104,105,106,107]; MWA is currently mainly applied for the treatment of HCC [108]. An example of percutaneous image-guided microwave ablation of an HCC nodule is represented in Figure 2.

#### 6.3.2. Immunomodulation

Compared to cryoablation and RFA, the immune response induced by MWA is relatively weak as evidenced by a significantly lower increase in IL-1, IL-6, and HSP-70 [71]. This may be the reason why the combination between MWA and immunotherapy has not been investigated in animal models as extensively as the other ablative methods [70]. One of the few studies on the matter, focused on breast cancer in mice [33], showed how combination therapy significantly prolonged survival and decreased the volume of tumors developing in animals after re-challenge. Combination therapy, compared to ablation monotherapy, also significantly increased the infiltration of CD8+ T cells into tumors, but not of CD4+ T cells. In HCC patients, immunohistochemistry analysis of biopsies taken before and after MWA [109] showed an increased infiltration of lymphocytes (mainly CD3+ T cells and CD56+ NK cells but not B cells) inside the ablated lesion, in the surrounding normal liver, and in distant untreated lesions. An inverse correlation was found between the density of infiltrates (of lymphocytes, macrophages, and CD56+ cells) into the ablated area and the risk of local recurrence. A phase I clinical trial in a small cohort of HCC patients with chronic HBV treated with a combination of MWA and immunotherapy reported an increase in CD8+ T cells one month after treatment and a reduction in HBV load [110]. Recently, a significant increase in circulating IL-12 (Th1 cytokine) and a decrease in IL-4 and IL-10 (Th2 cytokines) after MWA has been demonstrated, resulting in a positive antitumor response [111]. Therefore, although the antitumor immunity induced by MWA is weak and not sufficient alone to have a significant antitumor effect, the combination with immunotherapy to obtain a potential synergistic effect deserves further study.

A retrospective study on HCC patients showed no significant differences in VEGF and HGF receptors between patients treated with MWA and those treated with resection; on the other hand, in NSCLC, VEGF levels were found to be significantly increase after MWA [112,113].

## 7. Invasive Endovascular LRTs

### 7.1. Trans-Arterial Chemoembolization (TACE)

#### 7.1.1. Technique

**Figure 3:**
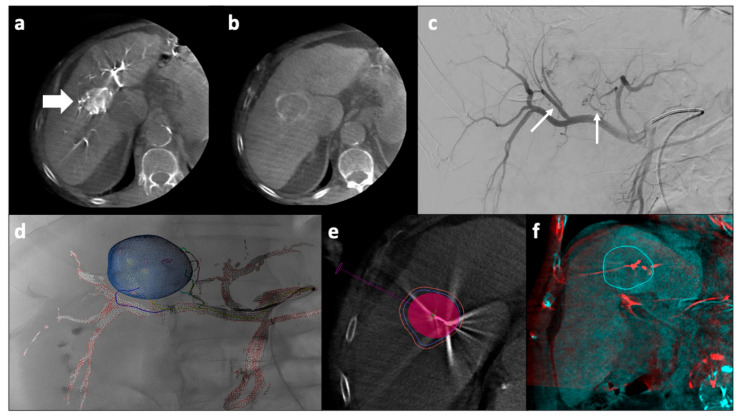
Percutaneous microwave ablation and trans-arterial chemoembolization for a large HCC nodule. For treating large single HCC nodules, a combination of ablation and embolization may be used. After arterial femoral access and catheterization of the celiac trunk and hepatic artery, a CBCT is performed that confirms the presence of a large nodule with arterial wash-in (**a**, arrow) and venous wash-out (**b**), consistent with HCC. The angiographic study demonstrated the presence of two main arterial feeders (thin arrows, **c**). Dedicated software can be used to delineate the target vessels and to plan the endovascular procedure (**d**). A dedicated software is also available to predict the ablation volume which is virtually created on a CBCT performed after having positioned the antenna inside the tumor, before starting the ablation (**e**,**f**). CBCT: cone-beam computed tomography; HCC: hepatocellular carcinoma.

TACE is the current standard of care for patients with intermediate-stage multinodular HCC [114]. Furthermore, in clinical practice, many patients in the earlier stage (i.e., single nodule or up to three nodules under 3 cm) carrying contraindications to curative approaches might be treated with TACE. The rationale for TACE is that the intra-arterial injection of a chemotherapeutic drug, such as doxorubicin, followed by embolization of the blood vessel, will result in a strong cytotoxic effect enhanced by ischemia. For large single tumors, a combination of ablation and embolization is used as illustrated in Figure 3.

#### 7.1.2. Immunomodulation

TACE and drug-eluting bead TACE (DEB-TACE) cause local cell death and induce a tumor-specific immune response [115]. For example, in a study by Kohles et al. [116], circulating GPC3-specific cytotoxic T lymphocytes (CTL) were shown to increase in 55% of patients with HCC after RFA and in 44% of patients after TACE.

Moreover, Treg count was reported to significantly decrease after TACE compared to baseline, and patients with a low post-TACE Treg count exhibited a significantly higher median time to progression (11.6 months) than patients with a high post-TACE Treg count (3.8 months) [117]. TACE causes changes in the levels of various cytokines: early increases in IL-6 after TACE indicate an acute-phase response and are correlated with post-treatment hepatitis, while late-phase increases in Th2 cytokine levels reflect immune suppression [118]. Moreover, Huang M et al. [119] reported that after treatment, CD4+ cells, CD4+/CD8+ ratio, and NK cells increased in HCC patients; in contrast, CD8+ cells were significantly reduced, offering a strong indication that TACE may improve patients’ immune system. 

Conversely, after TACE, surviving cancer cells may increase the expression of HIF-1 α and VEGF, which can lead to tumor progression [120,121].

### 7.2. Trans-Arterial Radioembolization (TARE)

#### 7.2.1. Technique

Yttrium-90 (90Y) microsphere radioembolization (TARE) is a promising modality that has emerged for the treatment of patients with advanced liver cancer in which resin or glass microspheres containing 90Y are administered directly into the hepatic arterial branches that supply the hepatic tumor [122]. TARE takes advantage of both radiation and embolization, although its effects are primarily mediated by radiation injury. 90Y microspheres, entrapped in the microvasculature of the liver, emit β-radiation mostly during the first 11 days following treatment, after which it decays into stable zirconium, resulting in tumor necrosis as a consequence of free oxygen radical generation and subsequent irreparable DNA damage, similar in principle to brachytherapy. Because of their small size (30–70 μm), radioactive microspheres are able to penetrate into the tumor vasculature with minimal embolic and hypoxic effects [123], being a valid option in patients with portal vein thrombosis. There is a high interest in the clinical use of TARE in locally advanced HCC because of the paucity of effective therapies in this group of patients [122]. This is the reason why, despite unsuccessful randomized control trials, this technique is widely applied. Patients who have failed TACE in early or intermediate stages of HCC, patients with multiple monolobular disease (>4 tumors), and patients with large tumors (>5 cm) and limited vascular invasion have been proposed as potential candidates for TARE [124].

#### 7.2.2. Immunomodulation

From the analysis of the immune profile of surgically resected HCC that had been downstaged by TARE, various signs of immune activation have been described, including higher intratumoral expression of GrB and infiltration of CD8+ T cells, CD56+ NK cells and CD8+ CD56+ NKT cells [125]. An increase in TNF-α in both CD8+ and CD4+ T cells was also observed after TARE as well as an increase in APCs, implying a systemic immune activation. A high percentage of PD-1/tim-3+CD8+ T cells co-expressing the homing receptors CCR5 and CXCR6 denoted TARE responders. A study on 25 patients with inoperable hepatic malignancies treated with TARE demonstrated that treated patients had a profound lymphopenia directly after therapy, whereas granulocytes and monocytes increased rapidly, leading to an overall increase in the total number of leukocytes; lymphopenia affected all subpopulations: CD3+, CD4+, CD8+ T cells, CD4+ CD8+ T cells, CD19+ B cells, and NK cells [126]. Finally, the intense immune activation following TARE was further confirmed by Fernandez-Ros N et al. [127], who demonstrated an increase in pro-inflammatory (IL-6 and IL-8) and oxidative stress (malondyaldehide) markers, an induction of endothelial injury markers (vW factor and PAI-1), an activation of the coagulation cascade (factor VIII, PAI-1, D-Dimer), and a significant increase in factors related to liver regeneration (FGF-19 and HGF). A significant increase in IL-1 and IL-6 levels after TARE was also described by Seidensticker et al. [128].

Compared to TACE, evidence suggests that the increase in HIF-1 α and VEGF obtained with TARE is significantly lower [129].

## 8. Ablation Techniques under Evaluation

### 8.1. High-Intensity Focused Ultrasound (HIFU)

HIFU is a non-invasive hyperthermic ablative modality by a multi-element ultrasound transducer, positioned outside the body or in a cavity that targets a focal area with high-intensity ultrasound beams. The convergence of these beams causes an increase in temperatures to approximately 60–85 °C, inducing tumor cells coagulative necrosis together with cellular apoptosis in the surrounding tissues [130,131]. HIFU has been used only in the last years for the treatment tumors [132]. Regarding HCC, Ji et al. reported CR and ORR rates of 66% and 83%, respectively, and a one-year survival rate of 81% by the analysis of several studies on HIFU in the treatment of HCC [133].

Regarding the potential immunomodulation effects of HIFU, in clinical settings, an increased expression of HSP-27, HSP-72, and HSP-73 was registered in prostate treatment, especially at the border of HIFU action [134]. In patients with solid tumors, including HCC, treated with HIFU, an increase in CD4+ lymphocytes and in the CD4+/CD8+ ratio was also reported [135], while serum immunosuppressive cytokines, in particular VEGF, TGF-β1, and TGF-β2, decreased [136]. More recently, an increased level of NK, CD3+, CD4+, CD8+, and CD4+/CD8+ was registered in patients with primary liver cancer at 3 months after HIFU when compared with the baseline; moreover, the levels of IFN-γ and IL-2 significantly increased, while the levels of IL-4 and IL-10 decreased, reflecting a change in the Th1–Th2 ratio [137].

A new HIFU application is named histotripsy in which extremely high pressure, very short (micro- or millisecond) acoustic pulses cause changes in gaseous tissue components, with bursting of bubbles, leading to mechanical fragmentation and transformation of tissue into a liquefied homogenate that can be reabsorbed by the body [138,139]. Pulses are separated by cooling intervals to avoid heat generation. By avoiding heat, histotripsy is not limited by the heat-sink effect, an advantage in highly vascularized organs like the liver, and leaves large vessels intact, as they have higher mechanical resistance to fractionation when compared to surrounding soft tissues [138,140]. For histotripsy, evidence of local and systemic inflammatory response has been reported in melanoma tumors in mice, with increased levels of intratumoral NK, DC, neutrophil, B and T cells, and increased levels of circulating NK cells; moreover, histotripsy led to inhibition of metastatic growth, and significantly higher levels of CD8+ T-cell infiltration into distant tumor sites were recorded after contralateral histotripsy ablation [141]. At a cellular level, release of DAMPs and an increased circulating level of HMGB1 were observed after histotripsy [141].

### 8.2. Laser Ablation

Laser ablation is a non-invasive technique in which laser optical fibers are focused into a target lesion, leading to temperatures of over 60 °C and causing coagulative necrosis. When applied in a minimally invasive modality to reach deep tissues, it is called laser interstitial thermotherapy (LITT) [142,143]. This technique is performed delivering light for 1–10 min, causing a photochemical damage on biological tissues with radical formation and inflammation and a thermal damage with denaturation of proteins.

In immune-modulating LITT (imILT), a temperature gradient is created in the tumor, with non-coagulating temperatures reached at the tumor border, which are kept for a longer time (30 min) in order to achieve ICD [144] with the release of DAMPs and antigens from the tumor margin [45]. As far as the available data on outcomes in HCC, a CR of 82–97% and cumulative three-year survival rates up to 73% have been reported in patients within Milan Criteria [145,146]. Another study reported a complete tumor ablation of laser-treated HCCs in 88% of cases, with complete ablation in 91.7% of nodules up to 5.0 cm [147].

For HCC lesions up to 5 cm in high-risk locations, a median survival of 3.5 years was registered [148]. When compared with RFA, no significant differences were found in terms of local control, OS, and safety by two randomized trials [149,150].

When considering the potential immunological effect of laser ablation, in mice models of liver cancer, LITT was followed by eradication of a subsequent challenging tumor and absence of tumor spread, and it was associated with an increased number of macrophages and CD8 T cells; expression of HSP70, HSP90, and HSP27; serum levels of IL-6 and TNF receptor [151,152,153]. In rat models with multiple implanted liver adenocarcinomas, the treatment for one tumor with laser ablation led to an increased expression of CD8, B7-2 (CD86), MHCII, LFA1 (CD11a), and ICAM1 (CD54) at the invasion front of another untreated tumor [154]. Conversely, another study showed that moderate (45 °C) heat stress of HCC and hepatocytes stimulated growth of non-heat-stressed HCC cells [155].

### 8.3. Stereotactic Body Radiation Therapy (SBRT)

SBRT involves the delivery of high doses of ablative radiation to limited (tumoral) liver volumes, with low risk of damage to surrounding tissues, by highly conformal hypo-fractionated external beam radiation in relatively fewer fractions compared to conventional radiotherapy [156,157]. 

In a meta-analysis including several studies, with a total of 7928 HCC patients, SBRT was well tolerated, had an OS equivalent to RFA, and was superior in terms of local control, especially for a diameter > 2 cm [158].

Regarding immunogenicity, SBRT causes cellular death and release of TAAs and DAMPs, such as HMGB1 [159], leading to an increased expression of MHC I molecules in a dose-dependent manner [160]. In radiation, cancer cell death occurs after DNA damage, favoring the release of double-stranded DNA that activates the cyclic GMP–AMP synthase/stimulator of interferon genes (cGAS/STING) signaling pathway, with the production of IFN-α and IFN-β, innate immunity stimulation, and lymphocyte infiltration in tumor tissue [161]. Pro-inflammatory cytokines are released with APC activation and migration to lymph-nodes, where specific CD8 T cells are activated [162,163]. 

Exposure to radiation in vivo promotes cell-surface expression of calreticulin in carcinoma cells, resulting in enhanced T-cell killing [164]. SBRT has been shown to have an effect on both peripheral NK and CD3+CD56+NKT-like cells, and higher percentages of the latter cell population was recently associated with increased OS in HCC patients [165]. However, at the same time, radiation therapy also has immunosuppressive effects. Interferon I receptor activity, which is enhanced by RT, has been associated to intratumor infiltration of Treg and myeloid cells and acquired resistance to anti-PD-1 monoclonal antibody [166]. The DNA damage response includes an increased expression of CTLA-4 and PD-L1 on the tumor cell membrane; PD-L1 causes exhaustion of T cells, which contributes to tumoral immune escape [167]. Moreover, Treg cells are more resistant to radiation than other lymphocytes, resulting in their preferential increase [168].

### 8.4. Irreversible Electroporation (IRE)

Irreversible electroporation is a percutaneous non-thermal ablation technique that delivers multiple short high-voltage electrical pulses, generating an electrical field that alters the electrochemical potential of the cell membrane and leads to the irreversible formation of nanopores in the lipid bilayer, causing necrosis and apoptosis [169,170]. As its main effects do not rely on high temperatures, IRE has the advantage over thermal ablative techniques of avoiding the heat-sink effect [171,172]. Moreover, by selectively destroying lipid bilayers, IRE preserves blood vessels and bile ducts due to their higher content of collagen and fibrous tissue [173,174]. On the other hand, IRE is technically demanding, costly, and more time-consuming than heat-based thermal ablation [175]. Furthermore, treatment requires general anesthesia, paralysis, and cardiac synchronization [176]; high-frequency IRE (HFIRE), the next generation of IRE that does not require cardiac synchronization and paralytic agents, could minimize these difficulties in the near future [177]. IRE has been generally used in HCC cases where thermal ablation was considered unsuitable or at high risk of complications including the setting of bridge to transplantation [178,179,180]. Regarding efficacy, a recent review of nine major studies focused on IRE in the treatment of liver cancers (the majority being HCC) reported a primary efficacy rate of 66–100%, a local recurrence rate of 5–34%, and general and major complication rates of 11–42% and 3–11%, respectively [181]. 

There is a growing interest in the immunomodulatory effect of IRE, which, unlike thermal ablative techniques, has the theoretical advantage of leaving intact tumor antigens within the ablated tissue and of preserving blood vessels and lymphatics, thus facilitating immune cells infiltration. Comparing the effects of IRE and RFA performed on mice with noncancerous liver, neutrophil and macrophage infiltration was higher after IRE within the ablation zone, along with intact microvessels; moreover, IRE led to greater cytokine expression, local inflammatory effects, and distant systemic effects [182]. A study on HCC in animals demonstrated that IRE ablation promotes infiltration of inflammatory cells adjacent to the ablation volume and release of several cytokines, reverting the abnormal Th2 status promoted by HCC (characterized by a decreased antitumor efficacy) back to a normal Th1 dominant status (which increases antitumoral activity) [183]. In HCC patients, the macrophage migration inhibitory factor (MIF), an immunomodulatory cytokine which maintains the inflammatory response, was increased more after IRE than after RFA; the same was observed for macrophage inflammatory protein-1b (MIP-1b)/chemokine ligand 4 (CCL4), TNF-α, and IL-17 [184].

## 9. Immunotherapy

### 9.1. Immune Checkpoint Inhibitors

PD1 and PDL1 inhibitors, such as nivolumab and pembrolizumab, have proven to be effective (ORR 15–20% with a 1–5% of complete responses, and prolonged survival) as both first and second line (after sorafenib) treatments of HCC in phase I/II studies, even though the data were not confirmed in terms of OS and PFS combined end-points in phase III studies [185,186,187]. Despite these inconclusive results, these drugs received accelerated approval by FDA as second-line treatments. To overcome these results, several strategies can be applied, such as the combination between CTLA-4 and PD1/PDL1, which increased response rates by 30%. In a phase II study, for example, the combination of ipilimumab and nivolumab obtained a median OS of 22.8 months [188] and was therefore approved to treat HCC patients after sorafenib by the FDA. Similar results were observed with the combination of the CTLA-4 inhibitor tremelimumab and the PDL1 inhibitor durvalumab in a phase I/II study [189].

The combination of pembrolizumab and lenvatinib has been tested in a phase Ib study, doubling the response rate compared to pembrolizumab alone, leading to a 20 months median overall survival, but at the cost of increased toxicity [190]. Another option is the association of PD1/PDL1 inhibitors with a TKI/anti-VEGF drug such as the association between atezolizumab with bevacizumab. This association proved to be superior to sorafenib at the primary analysis of the phase III study IMbrave150, with a median OS of 19.2 vs the 13.2 months of sorafenib; the HR was 0.58 (95% CI 0.42–0.79) (*p* < 0.001); median PFS (95% CI) per RECIST v1.1 by Independent Review Facility was 4.3 (4.0–5.6) months with sorafenib and 6.8 (5.7–8.3) months with atezolizumab + bevacizumab (HR 0.59, 95% CI 0.47–0.76; *p* < 0.001), and the overall response rates (per IRF RECIST v1.1) were 12% and 27%, respectively (*p* < 0.001). AE rates were similar in the two treatment groups (grade 3 or 4, 55% versus 57%; grade 5, 6% versus 5%, respectively) [191]. Given the efficacy and safety outcomes of the IMbrave150 trial, the combination of atezolizumab and bevacizumab was first approved by FDA on May 2020 and by the European Medicines Agency (EMA) on November 2020 for the first-line treatment of unresectable HCC.

### 9.2. Other Immunotherapies

A approach to modify the immune response to tumor microenvironment is represented by adoptive cell therapy (ACT), in which lymphocytes are isolated from the patients’ blood, expanded, and/or genetically engineered and then reinfused into the patient [192]. The most commonly used cells in HCC are lymphokine-activated killer (LAK) cells, cytokine-induced killer (CIK) cells, NK cells, TILs, and redirected peripheral blood T cells. Before the ACT treatment, cyclophosphamide and fludarabine are administered in order to obtain lymphodepletion and to support in vivo expansion of adoptively transferred cells.

Another potential approach are tumor vaccines, which are agents that are able to generate tumor-specific immune responses; however, data are still preliminary and inconclusive.

## 10. Combination of Immunotherapy-LRT

The immune activation against tumor induced by LRTs is not strong enough on its own to generate a clinically significant antitumor response and it is incapable of preventing tumor recurrence. From this derives the potential of combining LRTs with immunotherapy, in order to amplify and prolong the antitumor immune response instigated by the LRT, thereby reducing recurrence following ablation and improving outcomes.

The relationship between LRT and immunotherapy is therefore mutually beneficial and synergistic; on the one hand, LRT counteracts a key aspect of the tumor immunosuppressive status, namely, the immunosuppressive microenvironment and the lack of available tumor antigens, which undermines the efficacy of immunotherapy; on the other hand, immunotherapy enhances the immune stimulating effects obtained with LRT.

In fact, the immunological response after locoregional therapies seems to be magnified by checkpoint blockade and even more intensified by the add on of MKIs/anti-VEGF agents, both able of further remodeling the composition of the HCC microenvironment in favor of an antitumoral effect inducing the transformation of a non-immunogenic “cold” into an inflamed “hot” tumor [40,41,42].

When exploring the combination of ICIs with intravascular treatments, such as TACE or TARE, the target population should be the BCLC B stage with well-preserved liver function, and the control arm should be TACE alone. There are some aspects that should be considered that can potentially limit the results of these trials, mainly the controversial definition of primary endpoints and the heterogeneity of both BCLC B population and TACE procedures. Because of this background noise, stratification in terms of tumor burden, endovascular techniques applied, and AFP levels are of paramount importance in the interpretation of results. After the negative results with sorafenib in combination with TACE, several phase II trials are underway, but the available data is still not strong enough to be considered as reliable; at this time point, they suggest that combining systemic therapies and locoregional therapies with ICIs may represent a future useful strategy to enhance the results of locoregional therapies in the intermediate stage population [45]. Regarding curative treatments, the most important point to remark remains the fact that 70% of patients develop hepatic recurrence at 5 years, negatively impacting the overall prognosis. Many adjuvant strategies, including sorafenib, have failed to improve relapse-free survival (RFS) or OS, but following the positive results on RFS in the adjuvant setting for several other tumoral conditions and the knowledge we are building on immunotherapy and HCC, we are now testing the rationale of its application in this setting.

The mechanism behind the combination of ablative procedures with immunotherapy is somehow different; ablation not only induces the release of tumor antigens, but it also increases the release of inflammatory cytokines, stimulating an antitumor systemic immune response, even more enhanced if it is followed by an adjuvant immunotherapy.

The concept that BCLC stage 0/A patients with well-preserved liver function should be the population target of trials testing immunotherapy in the adjuvant and neoadjuvant setting is widely shared, and so it is the belief that the primary endpoint should be represented by RFS/time to recurrence. As a result of this, stratification criteria should be taken into consideration, such as size and number of lesions, region of origin of patients and other risk factors of recurrence (i.e., microvascular invasion at histology or AFP levels before locoregional treatment/surgery).

Another potential combination of LRT and immunotherapy is in advanced HCC, where thermal ablation has not its typical curative aim, but its role would be to increase the effectiveness of immunotherapy by transforming a “cold” tumor into a “hot” one. Duffy et al., the first to report this approach, combined tremelimumab with either TACE (BCLC B stage) or subtotal ablation (BCLC C stage) in a cohort of patients who had progression or were intolerant to sorafenib, and achieved a partial response rate of 26.3% (*n* = 5/19 patients) when measured on lesions outside the ablation or chemoembolization zone, a median time to progression of 7.4 months and a median OS of 12.3 months; moreover, the majority of patients with HCV showed a significant reduction in viral load [193].

Currently, several studies are underway evaluating the combination of nivolumab with SIRT (NCT03380130, NCT03033446, and NCT02837029), pembrolizumab with SIRT (NCT03099564), nivolumab with TACE (NCT03143270 and NCT03572582), pembrolizumab with TACE (NCT03397654); ongoing trials are presented in Table 2.

In a recent study focused on the combination of RFA and cellular therapy in HCC, mononuclear cells were harvested and induced into NK cells, γδT cells, and CIK cells, which were infused back into the RFA-treated patients. The combination between these immune cells and RFA improved progression-free survival and reduced HCC recurrence compared to RFA alone [194].

Regarding the safety of LRTs combined with immunotherapy, the available data seem to point towards an acceptable safety profile; for example, TARE plus nivolumab showed a similar safety profile when compared to TARE alone [195], tremelimumab in combination with ablation proved to be safe for the treatment of advanced HCC [193], no significant adverse reaction was found when combining RFA with cellular immunotherapy in HCC patients [194].

## 11. Conclusions

Interventional radiology image-guided LRTs have an established role in the treatment of patients with HCC. Immunotherapy has become an important part of the current available therapeutic options for HCC in the last years. HCC is now known to be characterized by changes in the immune system, including immunosuppression and immune evasion. The different types of LRTs not only cause destruction of tumor cells, but also have immunomodulatory effects, which have been demonstrated both in preclinical and clinical studies. The combination of immunotherapy and LRTs could lead to mutually beneficial effects. On one hand, immunotherapy could enhance the immunostimulatory effects of LRTs, thus reducing recurrence rates after their application; On the other hand, the use of LRTs, also in advanced stages of disease, by altering the original tumoral immune statu, could increase the efficacy of immunotherapy and, potentially, the number of patient treatable with systemic immunotherapy. The importance of this new approach to the disease is demonstrated by the number of ongoing clinical studies focused on this combination, which could open a new chapter in the treatment of HCC.

## Figures and Tables

**Table 1:** Summarized immunological effect of LRTs.

	RFA	Cryoablation	MWA	TACE	TARE	HIFU	Laser	SBRT	IRE
**Increased**	DAMPs (RNA, DNA, HSPs, HMGB1) Inflammatory cytokines (IL-1β, IL-6, IL-8, TNF-α, IFN- γ) Tumor specific antibodies CD4+ T, CD8+ T, tumor-specific T, cytotoxic T, central memory lymphocytes, infiltrating CD45RO+	Inflammatory cytokines (IL-1, IL-6, TNF-α) NF-κβCD4+ T, CD8+ T, NK cells	DAMPs (HSP-70) Inflammatory cytokines (IL-1, IL-6, IL-12) CD3+ T, CD56+ NK, CD8+ T cells)	Circulating GPC3-specific cytotoxic T lymphocytes (CTL) IL-6 CD4+ cells CD4+/CD8+ ratio NK cells	Inflammatory cytokines (TNF-α, IL-1, IL-6, IL-8) CD8+ T cells CD56+ NK, CD8+ CD56+ NKT, CD4+ T cells APCs oxidative stress markers (malondyaldehide) endothelial injury markers (vW factor, PAI-1) liver regeneration factors (FGF-19, HGF)	DAMPs (HSPs, HMGB1) Inflammatory cytokines (IFN-γ, IL-2) CD4+, CD8+, CD3+, NK cells, B cells CD4+/CD8+ ratio DC Neutrophil	DAMPs (HSPs) Inflammatory cytokines (IL-6) Macrophages CD8 T cells	DAMPs (HMGB1)MHC I molecules Inflammatory cytokines IFN-α, IFN-β Lymphocyte infiltration in tumor tissue Specific CD8 T activation Peripheral NK, and CD3+CD56+NKT-like cells Treg	Neutrophil and macrophage infiltration Inflammatory cytokines MIF Macrophage inflammatory protein-1b (MIP-1b)/chemokine ligand 4 (CCL4), TNF-α, and IL-17
**Decreased**	TGF-ß, IL-10 Tregs		IL-4, IL-10	Treg CD8+ cells		Immunosuppressive cytokines (VEGF, TGF-β1, TGF-β2 IL-4, and IL-10)			

DAMPs: danger-associated molecular patterns; HSPs: heat shock proteins; HMGB: high-mobility group box; GPC3: glypican-3; APCs: antigen--presenting cells; vW: von Willebrand; PAI-1: plasminogen activator inhibitor-1; FGF-19: fibroblast growth factor-19; HGF: hepatocyte growth factor; DCs: dendritic cells; MIF: macrophage migration inhibitory factor; MIP-1b: macrophage inflammatory protein-1b; MHC: major histocompatibility complex.

**Table 2:** Trials on LRT and immunotherapy combinations.

Clinicaltrials.gov ID	LRT	Immunotherapy	Phase	Line of IO	Study Design	Disease Stage
02568748	TACE	CIK	III	Adjuvant	Open label	BCLC B
03592706	TACE	Immune killer cells	II/III	Sequential	Randomized	BCLC B, C
03638141	DEB-TACE	CTLA-4/PD-L1 (Durvalumab and Tremelimumab)	II	Sequential	Open label	BCLC B
03572582	DEB-TACE	Nivolumab	II	Combination	Open label	BCLC B
03937830	DEB-TACE	Durvalumab, Tremelimumab	II	Combination	Open label	BCLC B, C
03575806	TACE	Autologous Tcm immunotherapy	II (completed)	Sequential	Open label	Child–Pugh A
02487017	TACE	DC-CIK	II	Combination	Open label	Child–Pugh A, B
02856815	TACE	CIK	II	Adjuvant	Open label	BCLC B
03397654	TACE	Pembrolizumab	IB	Sequential	Open label	Child–Pugh A
03143270	DEB-TACE	Nivolumab	I	Combination	Open label	BCLC B
03817736	TACE and SBRT	ICI	II	Sequential	Open label	Child–Pugh A, B
03124498	TACE, RFA, PEIT	CIK	I/II	Adjuvant	Open label	Child–Pugh A, B
02821754	TACE, RFA, cryo	ICI (Durvalumab, Tremelimumab)	II	Combination	Open label	BCLC B, C
01853618	TACE, RFA	Tremelimumab	I/II (completed)	Combination	Open label	BCLC B, C
03383458	Ablation	Nivolumab	III	Adjuvant	Randomized	Child–Pugh A
03380130	TARE	Nivolumab	II (completed)	Sequential	Open label	Child–Pugh A
02837029	TARE	Nivolumab	I	Combination	Open label	Child–Pugh A, B
03033446	TARE	Nivolumab	II	Combination	Open label	Child–Pugh A
03099564	TARE	Pembrolizumab	I	Combination	Open label	Child–Pugh A, B
03259867	TATE	Nivolumab or Pembrolizumab	IIa	Combination	Open label	BCLC C

Abbreviations: CIK: cytokine-induced killer cell; DC: dendritic cell; DEB-TACE: drug-eluting bead-trans-arterial chemoembolization; PEIT: percutaneous ethanol injection therapy; RFA: radiofrequency ablation; SBRT: stereotactic body radiation therapy; TATE: trans-arterial tirapazamine embolization.
